# Influence of gender roles and rising food prices on poor, pregnant women’s eating and food provisioning practices in Dhaka, Bangladesh

**DOI:** 10.1186/1742-4755-10-53

**Published:** 2013-09-26

**Authors:** Adrienne V Levay, Zubia Mumtaz, Sabina Faiz Rashid, Noreen Willows

**Affiliations:** 1 School of Public Health, 3–309 Edmonton Clinic Health Academy, University of Alberta, 11405 – 87 Ave, Edmonton AB T6G 1C9, Canada; 2 James P. Grant School of Public Health, 5th Floor (Level-6), icddr,b Building, BRAC University, 68 Shahid Tajuddin Ahmed Sharani, Mohakhali, Dhaka 1212, Bangladesh; 3 Department of Agricultural, Food & Nutritional Science, Edmonton Clinic Health Academy, University of Alberta, 11405-87 Ave, Edmonton AB T6G 1C9, Canada

**Keywords:** Maternal malnutrition, Pregnancy, Gender, Women, Food insecurity, Urban, Ultra-poor, Bangladesh

## Abstract

**Background:**

Maternal malnutrition in Bangladesh is a persistent health issue and is the product of a number of complex factors, including adherence to food 'taboos’ and a patriarchal gender order that limits women’s mobility and decision-making. The recent global food price crisis is also negatively impacting poor pregnant women’s access to food. It is believed that those who are most acutely affected by rising food prices are the urban poor. While there is an abundance of useful quantitative research centered on maternal nutrition and food insecurity measurements in Bangladesh, missing is an understanding of how food insecurity is experienced by people who are most vulnerable, the urban ultra-poor. In particular, little is known of the lived experience of food insecurity among pregnant women in this context. This research investigated these lived experiences by exploring food provisioning strategies of urban, ultra-poor, pregnant women. This knowledge is important as discussions surrounding the creation of new development goals are currently underway.

**Methods:**

Using a focused-ethnographic approach, household food provisioning experiences were explored. Data from participant observation, a focus group discussion and semi-structured interviews were collected in an urban slum in Dhaka, Bangladesh. Interviews were undertaken with 28 participants including 12 pregnant women and new mothers, two husbands, nine non-pregnant women, and five health care workers.

**Results:**

The key findings are: 1) women were aware of the importance of good nutrition and demonstrated accurate, biomedically-based knowledge of healthy eating practices during pregnancy; 2) the normative gender rules that have traditionally constrained women’s access to nutritional resources are relaxing in the urban setting; however 3) women are challenged in accessing adequate quality and quantities of food due to the increase in food prices at the market.

**Conclusions:**

Rising food prices and resultant food insecurity due to insufficient incomes are negating the recent efforts that have increased women’s knowledge of healthy eating during pregnancy and their gendered empowerment. In order to maintain the gains in nutritional knowledge and women’s increased mobility and decision-making capacity; policy must also consider the global political economy of food in the creation of the new development goals.

## Background

Malnutrition is one of the most important risk factors for poor health, both directly and indirectly [1]. In Asia, an estimated 563 million people are malnourished [2] and young women of reproductive age are considered to be among the most vulnerable to malnourishment [3,4]. While the issue of malnourished women is problematic all the time, it is particularly so when pregnancy occurs.

Bangladesh continues to struggle with levels of maternal malnutrition that are among the highest in the world. Almost one-quarter [5] of Bangladesh’s 81 million women [6] are considered to be undernourished with a body mass index (BMI) of less than 18.5 [5]. Not only is there a negative impact on children born to malnourished mothers, but there is also an increased risk of maternal morbidity and mortality [3].

A large body of literature lists causes of these high rates of maternal malnutrition. They include women’s lack of knowledge regarding biomedically recommended pregnancy dietary requirements [7] and adherence to traditional beliefs and practices that limit certain types of 'nutritious’ foods [8-10]. For example, humoral beliefs that classify foods as 'hot’ or cold’ are found to impact pregnancy eating practices. Pregnancy is understood to be a 'hot’ condition where 'hot’ foods should be avoided and 'cold’ foods eaten. In most settings, adhering to these beliefs can lead to exclusion of highly proteinaceous foods like, in Bangladesh, fowl, fish, and lentils [11,12].

Another important factor listed as a cause of maternal malnutrition is the gender order of Bangladesh [13,14]. In this patriarchal society, the subordination of women is systematically built into the societal structures [13,14]. Limitations on women’s decision-making, dependency and mobility, as dimensions of the gender order, disables them from being involved in food provisioning [11]. Although the practice of *purdah* (keeping women in seclusion) has never been strictly adhered to in Bangladesh, women are still 'deterred’ from going out in public after dark as well as many other public "male" spaces during the day time, like the *bazaar* (market) [15]*.* Women in Bangladesh, particularly in rural areas, often have no control over how the household food budgets will be spent [12]. The gender order also disadvantages women through gendered food allocation that often privileges males [16], an inequitable distribution of food that can begin in childhood [17]. This leads to women’s lifelong poor nutritional status, particularly when combined with multiple pregnancies and strenuous workloads [18].

We argue an additional important factor that may impact the prevalence of maternal malnutrition is food insecurity [19,20]. Food insecurity has been defined as a condition that exists "when people do not have adequate physical, social or economic access to food" [21]. Bangladesh’s level of food insecurity has been profoundly impacted by the global food price crisis of 2008. In the period from 2010 to 2011 alone, the price of food in the country increased by 19% effectively preventing poorer groups from accessing an adequate amount of food [22]. The food price crisis was a culmination of a complex melange of factors which converged to create an environment in which prices of food rose dramatically. Some of these factors include increasing oil prices; land being converted to growing corn to meet the biofuel targets of western nations; increasing demand for meat products from massive emerging economies; deregulation of the agricultural industry and the concentration of global food production into the hands of only a few agricultural corporations; agricultural subsidies in western nations leading to cheap food aid thereby eradicating the demand for local farmers to grow food; market speculation; and globally decreasing grain stores [23,24]. The people most vulnerable to food price hikes in Bangladesh are poor urban dwellers who are dependent on the market for their food needs [25], earn inadequate incomes [26] and spend up to 70% of their daily income on food purchases [27]. Food insecurity among this particular group of people in Bangladesh is almost universal [26].

However, despite this evidence much of the research in developing countries, including Bangladesh, has remained focused on simply measuring nutritional statuses using quantitative anthropometric measures[28-30]. This research has also centered on micronutrient deficiencies and micronutrient supplementation and the impact of these on maternal and child health outcomes [31-33]. A smaller but emerging body of literature has addressed food insecurity in Bangladesh by either investigating the seasonality of food insecurity [29], food production [34] or attempted to develop food insecurity measures using quantitative scores[28,30].

Missing in this body of quantitative literature is an understanding of how food insecurity is experienced by people who are most vulnerable, urban ultra-poor women. Only one study to-date has mapped the experiences of food insecurity amongst ultra-poor, marginalized women in Bangladesh [26]. In their sample of 40 women who were the heads of their households, food insecurity was universal, with inadequate incomes being the key reason identified by the authors. While this study investigated female-headed households amongst Bangladeshi women, the experiences of pregnant women are unknown. What are urban poor, pregnant women’s perceptions of a healthy diet during pregnancy? What factors allow them to actualize their knowledge into practice? What are the barriers? Exploring the experiences of food insecurity among ultra-poor pregnant women, a largely invisible population, is important not only directly for the health of women themselves but also for the health of the next generation[35-37].

McIntyre *et al.*[26] has proposed that interrogating household food provisioning strategies is one promising method for acquiring an in-depth understanding of lived experiences of food insecurity This approach has the potential to elucidate how key factors such as personal and household resources, multiple priorities, social, political and cultural issues influence everyday household food provisioning experiences [38]. Furthermore, exploring food provisioning strategies has the potential to offer context-specific insight into experience that may not otherwise emerge through quantitative food security or nutritional status indicators[39]. There is a need for more "local" understandings of food provisioning experiences, particularly those of the most vulnerable groups. This is important because "the ultra-poor are not a homogenous group (p.27)" [40] and their voices must be heard [41] before real and sustainable solutions can be found [42].

The purpose of this study was to explore food provisioning experiences of poor pregnant women in Bangladesh in a context of urban poverty, an inequitable gender order and rising food insecurity. Most of the research to-date has been undertaken in rural Bangladesh. There is a paucity of literature examining the food provisioning experiences of urban women. A deeper understanding of these women’s experiences through personal narratives are necessary, particularly as discussions regarding the formulation of new development goals are underway to replace the unachieved Millennium Development Goals [43,44].

## Methods

### Setting

Fieldwork occurred from September 2010 to January 2011 in an urban, resource-deprived settlement ("slum" or *bastee*) in Dhaka, Bangladesh. Built on government land without government approval and therefore largely denied government resources [45], domiciliary units were built of corrugated tin and bamboo, set amidst open-sewers and poor sanitation. Some units had illegal electricity connections and well-water, installed by non-governmental organizations (NGOs). NGOs had a large presence in this *bastee* and had established primary schools, advocacy services, health services, as well as the installation of infrastructure such as deep tube-wells and squatter toilets.

The inhabitants of this slum were mostly engaged in low-paid, labour- intensive work in the informal sector such as bicycle rickshaw drivers and day labourer positions. Rural migrants that live in *bastees* largely lack the specific skills and educational background necessary to gain employment in the formal sector [45,46]. With that being said, it must be kept in mind that this particular *bastee* was heterogeneously comprised of rural migrants, those who were born there, landlords, garment workers and low-level government employees, to name a few. The location of the site, nestled between two wealthy neighbourhoods, made it extremely precarious for the inhabitants as developers eagerly desired to appropriate this land. In fact, during the writing of this paper, a portion of this slum was aggressively razed by the government for this purpose [47].

### Recruitment and sampling

The study population included currently pregnant women, women who had been pregnant within the last year and community members, including older mothers, mothers-in-law, landladies, neighbours, *dais* (traditional birth attendants), skilled birth attendants and husbands. Pregnant women and new mothers were chosen to garner insight into their perceptions of their food provisioning experiences. Relatives, community members, and delivery attendants were selected to garner a sense of the social environment in which urban poor pregnant women exist. We were initially introduced to potential participants by a key informant, a long-time inhabitant of the *bastee*. Purposive snowball sampling was used following these initial meetings to recruit subsequent participants [48]. We also met potential participants by wandering around the market on-foot, introducing ourselves to women with newborn babies. Rapport was built through multiple visits with participants and their families and neighbours, including husbands, mothers, mothers-in-law, fathers, children, and landladies. Space was given to allow conversations and interactions to evolve naturally. Furthermore, to gain trust from participants, we attempted to create distance between ourselves and the large local NGO that had a significant presence in this slum by reassuring participants we were not directly associated with this group. Participants held this NGO with some distrust. In the end, 12 pregnant women and new mothers, five delivery attendants, nine community members and relatives, and two husbands, for a total of 28 participants were recruited.

### Data collection

Using a focused-ethnographic approach, data were collected using one-on-one in-depth interview, focus group discussion and participant observation methods. In-depth interviews took place in participants’ dwellings. The Primary Investigator (PI) led semi-structured interviews with participants using the translation services (linguistic and cultural) of the Research Associate (RA), a native speaker and Anthropologist. After explaining the purpose of the research to potential respondents, verbal consent was garnered. Participants were asked to provide socio-demographic information including household income, occupations of the household members, years married, migration status, and number of years in this slum. Socio-demographic information was recorded in the field notes and interviews were digitally recorded then translated and transcribed by the RA in collaboration with the PI.

Participants were then asked to describe their typical day from beginning to end, including all activities, and to give an informal food recall for the day. Those activities pertaining to types of food, food preparation and food provisioning were probed further to elicit more information, including food costs and allocation and decisions made. Also, respondents were asked to describe what they knew to be a healthy pregnancy diet and any traditional beliefs regarding food and pregnancy, to ascertain whether these beliefs played a role in women’s food provisioning decisions. Participants received a small token of gratitude for their time such as a *sharee* or a shawl of the style commonly worn in the *bastee*.

One focus group discussion was conducted much in the manner of the interviews but with the added benefit of multiplicative knowledge [49]. Also, questions and reflections that emerged during in-depth interviews were discussed. More focus groups had been planned but due to the duration of the fieldwork (master’s level research project) and unforeseen circumstances, only one could be arranged.

Participant observation throughout the fieldwork resulted in daily detailed field notes which aimed to contextualize the data. Pregnant respondents and other community members allowed us to accompany them to purchase their daily food from the market where multiple informal interactions also occurred with other community members, including food vendors. Furthermore, we spent an entire day with four of the pregnant respondents we interviewed.

### Analysis

Data were analysed via a latent analysis to ensure that it was not being analyzed in a manner detached from its context [50,51]. The steps of analysis were interrelated and carried out in conjunction and in reverse as it is a continual and non-linear process to help ensure confirmabilty [52] as well to have the opportunity while in the field to address any unclear ideas or questions with colleagues in Bangladesh and with participants. Coding was conducted in the following manner: (1) repeated in-depth readings of the transcripts, (2) in-depth reading while open coding, (3) organizing of codes into categories, (4) organizing categories into domains. Reflection on the domains led to development of themes. Commonalities between participants’ narratives were analysed while variations were also taken into consideration. Peer debriefing was done throughout the analysis to ensure credibility [50,53,54]. To ensure dependability, memoing was used throughout the research process, documenting any decisions regarding data collection and analysis [54,55]. Reflexive journaling was also conducted daily by the PI, ensuring her location in the research process remained clear and transparent.

Ethics approval for this study was granted by the James P. Grant School of Public Health at BRAC University in Dhaka, Bangladesh and the Health Research Ethics Board Panel B (HREB) at the University of Alberta in Edmonton, Canada.

## Results

### Participant characteristics

Table 1 outlines the pregnant respondents’ relevant characteristics. Ages of the pregnant or recently pregnant participants ranged from 16–30 years and none of them had more than two children at home. Of the five healthcare workers, three were *dais,* one a community health worker, and one a skilled birth attendant. Overall, while a few of the participants were born and raised in Dhaka, most were rural migrants with some having recently come from the rural area and others having been in Dhaka for over 20 years. All the names of the participants have been changed.

**Table 1:** Characteristics of 12 pregnant or recently pregnant respondents

**Name**	**Age**	**Months pregnant**	**Number of living children**	**Highest level of education attained (class)**	**Rural migrant? (Y/N)**	**Husband’s occupation**	Estimated reported household daily income from husband’s work (BDT)^*^
Jorina	28	9	2	7	Y	Tea vendor	150-200
Rokeya	22	8	0	2	Y	Rickshaw puller	200
Assia	22	2	0	n/a	Y	Water delivery	n/a
Ruma	20	new mother	1	5	N	Tailor	150
Jasmine	18	7	0	8	Y	Fish merchant	100-150
Mony	25	8	2	0	Y	Rickshaw puller	250
Jinna	18	new mother	0	3	Y	Rickshaw puller	100-150
Moklima	30	8	2	0	Y	Moori wallah	60-80
Mokta	19	3	0	n/a	Y	Rickshaw puller	100-150
Parul	19	4	0	0	Y	Rickshaw puller	130
Reshmi	16	5	0	0	Y	Rickshaw puller	100-150
Runa	18	7	1	0	Y	Hotel staff	200

^*^At the time of data collection in 2010, 71 BDT = 1 CAD.

### Findings

Three themes emerged from our findings: the dominance of biomedical knowledge of healthy eating practices while pregnant, the changing gender order, and rising food prices impacting food provisioning practices.

### Women know what to eat

The first theme that emerged was that all women knew the importance of healthy eating in pregnancy to ensure a healthy pregnancy and childbirth. Participants’ knowledge of what to eat, according to general biomedical recommendations [7] to ensure a healthy pregnancy and childbirth, was universal. The women reported that they had been told to eat more food while pregnant, to eat more fish, milk, eggs and vegetables as well as to take vitamin pills. Many received this nutrition counselling from the local delivery centre staff.

Pregnant women reported receiving advice/knowledge/information from a number of different sources including NGO health workers, *dais*, family members, landladies, neighbours, and doctors. Despite the omnipresence of NGOs in this slum providing knowledge about eating practices while pregnant, traditional sources, such as older women and family members, continue to be deemed important sources. Most importantly, though, these more traditional sources gave information that was also not divergent from bio-medically-based knowledge provided by doctors and NGO health workers. Women were advised by older women to "*to eat more vegetables and take more water"*. *Dais* also reported that they advised pregnant women that they should be eating meat or fish and increase vegetable intake:

*"We tell them that if they can get meat or fish they should eat these on a daily basis. Those who are rich, I tell them to eat meat and fish. Those who are poor, like me, I suggest them to eat peas and low-priced fishes a like shing fish…and also vegetables*." (Laila Begum, *dai*)

Food taboos did not emerge as an important predictor of eating practices. While women knew of taboo foods, for most it was an amusing subject when it was brought up by the research team. None reported adhering to these taboos, although probing indicated that most women avoided one type of fish, *mrigal maach*, because of their belief it might cause *mrigy* (epilepsy) in the baby.

*"They suggest not taking eels, baing fish and duck. If anyone takes duck, then the voice of her baby will be like the voice of a duck (laughs). They suggest to take sufficient fruits but not to take grapes. Grapes make the body hot…. I ate all of these things in pregnancy, even duck. My daughter is perfectly alright…in this pregnancy I am also taking all kinds of foods."* (Runa, 7 months pregnant)

### Relaxing gender norms

The second important theme that emerged was that normative gender rules responsible for restricting women’s mobility, visibility and decision-making capacity in Bangladesh are relaxing in the slum.

#### Increased mobility and decision-making in the slum

Our observations showed that many women walked "freely" in public within the *bastee’s* boundaries, including when shopping for food at the large *bazaars* within the *bastee* that were in close proximity to their homes. However, when women left the *bastee* they reportedly wore a *burqa*. This indicates the *bastee* was perceived as an 'inside space’ where everyone was viewed as a kin-relation and where it was acceptable for women to "*go to bazaar without any rigid veil*" (Assia, 2 months pregnant)

The participants contrasted their mobility in the urban area with the more restrictive mobility norms of the rural area and how this had impacted household food provisioning; shopping in the *bazaar* was acceptable for women in the *bastee*, while it was unacceptable in rural areas.Similarly, women had a say in what was purchased. This increased decision-making control regarding food purchases is exhibited by one woman who claimed that: '*most of the time he buys bazaar items depending on his desire…my husband prefers to buy fish and he does not like vegetables. But in my pregnancy period I need vegetables. At my request he will purchase vegetables."* (Rokeya, 8 months pregnant)

"*I usually do bazaar…[my husband] cannot manage the time for shopping…There is no problem at all with me going to bazar alone.*" (Mokta, 3 months pregnant)

"*In the rural area my husband did the bazaar because in the rural area women do not go to bazaar. The men restrict the women from going to bazaar. They like to keep the women in the veil always*" (Mony, 7 months pregnant).

It is evident, then, that women, in the clear articulation of the difference in mobility, visibility and decision-making in the rural versus urban area, were aware of these 'relaxing’ gender norms. Doing *bazaar* and having control over food purchases may play a role in determining the quality and quantity of food being purchased within poor, pregnant women’s households [56]. The women in this study were largely responsible for either purchasing the food or telling their husbands what to purchase, including more expensive items that the women desired. Women often are denied this control if living with her marital family as these types of decisions are within the realm of the mother-in-law [12]. However, as discussed in more detail below, there is a well acknowledged trend towards nuclear family structures amongst rural to urban migrants which exclude mother-in-laws [57-59].

It is worth mentioning that not all women were happy with these relaxing gender norms. The *bazaar* was still viewed as a 'dangerous place’ for women by men and women alike. Women still attempted to adhere to dominant gendered norms while out in 'public’, like ensuring head and abdomens were covered. Most reported they did not enjoy this increased freedom, with some continuing to believe that being out in public is a shameful activity for women.

#### Non- traditional family structures in slum

Normative gender rules generally decree women cohabitate with her marital family (virilocality). Contrary to norms of virilocal residence in Bangladesh, many of the women in this study were living in a nuclear family structure with natal family members living close by; in one case a woman’s natal family was living next door to her and her husband. This largely happened because only young men migrate alone while young unmarried women come with their families who, more often than not, find husbands amongst these young solo men. Women’s access to adequate amounts and types of food may be improved by this transformation of family structures in this context. According to the respondents, married women living with their natal families will usually have all of their expenses paid for by them, including food. The woman who had her natal family living next door, recalled eating an amount of food that was more substantial than participants whose families were not living in the *bastee*:

*"[For breakfast] I ate in my mother’s house…pumpkin and prawns. Besides that, in my house I cooked fish with potatoes and also* shutke (*dried fish*) *with* kochu (*a green local vegetable*)…*I desired to eat pumpkin. My mother cooked it yesterday night so she called me over to eat pumpkin curry this morning*." (Rokeya, 8 months pregnant)

The practice of walking over to their mother’s house for food, or sending a child over to grandma’s for food for that matter, was common.

Despite these relaxing gender roles, some women still do eat last in their household. However, this was only reported by one participant who was living with two children and her husband:

*"I sit last for food…I have to feed my children by my own hand."* (Jorina, 9 months pregnant).

The practice of women feeding their children by hand at every meal is widely practiced in Bangladesh, even among middle and upper class families. This may potentially create, in poorer households, issues of unequal food distribution where there simply may not be enough food to fulfill the desires or nutritional requirements of the entire household. However, because in our field site most women were living in nuclear family structures and were pregnant with their first child, this did not appear to be an issue.

### Insufficient incomes and rising food prices: double burden

The third theme emerging from the findings of this study is that dimensions of poverty, including rising food prices and insufficient incomes, is universally impacting poor, urban Bangladeshi women’s access to food.

#### Income poverty

As per table 1, all of the pregnant women’s husbands were involved in low paying occupations, such as bicycle rickshaw pullers, tea vendors (selling tea at 4BDT a cup, or 0.06 CAD) or tailoring. These occupations earned an estimated 60–250 BDT (0.84- 3.52CAD) per day. All of the incomes from their husband’s work in Dhaka were from daily wage labour, characterized by irregularity and uncertainty. It was also reported numerous times that husbands were regularly gambling with these daily incomes.

None of the women (with the exception of two who worked part-time as domestics, adding an extra 60BDT (0.84 CAD) of income per day to the household income) were employed. The majority of pregnant respondents did report employment prior to marriage or prior to pregnancy. Reasons given by participants as to why they had left their jobs after becoming pregnant were fear of being injured in the workplace and in other public places, weakness, husbands requiring they leave work, and illness while pregnant.

Reported expenditures on daily food provisions ranged from 100–150 BDT, about 50% to 100% of their daily household incomes. From participant observations in the *bazaar* and accompanying women making food purchases, it was observed that this amount bought rice and vegetables, oil and spices, and perhaps, on a more lucrative day, some *choto maach* (small fresh fish) or *shutke* (dried fish) to feed a family of two to four.

All but one of the pregnant women had migrated from the rural area where their families had been living in impoverished conditions as landless tenant farmers. One older woman we interviewed even claimed that her family migrated to Dhaka because the island she was from eroded into the river. Evidently, while some women reported that they preferred Dhaka to the rural area because there were more opportunities for employment for their entire family, for most the quality of life had not improved significantly as settlement dwellers endured inadequate incomes, rising food prices, and hostile living conditions.

#### Rationing of food

One impact that insufficient incomes had on eating practices was that pregnant women were driven to rationing household food:

*"[My husband] did the shopping yesterday…if I cooked it at morning time today then there would be nothing left for supper today."* (Moklima, 8 months pregnant)

The above response was given by a pregnant woman when asked why she had eaten only *panta bhat* (rice water gruel) for breakfast that morning. While food rationing was occurring, none of the women reported missing a meal.

#### Here you have to buy everything

Women who had migrated from rural areas compared their food-provisioning experiences between urban and rural areas. They felt they struggled to acquire sufficient foods because they had to purchase everything from the market. This, in combination with insufficient incomes and rising food prices, critically impacted women’s eating practices. One older *dai* claimed that acquiring high quality and quantities of food was easier in rural areas and that the challenges faced by poor urban migrants in food acquisition was impacting pregnant women’s nutrition in the urban slums:

"*Long before, we took our meal fresh and hygienic and we had no want of fresh foods. We collected fresh milk, fish and other foods from the land and those were fresh and hygienic. But this is not possible in the city. The city dwellers are able to get fresh food only half the days of the week. So the pregnant women suffer from malnutrition and they feel weak and [the dais] can do nothing for them."* (Beauty, 65 years old, *dai*)

This sentiment was echoed by another young, pregnant woman who not only commented on the quality of food but also the quantity:

"*There are some difference between Dhaka and the rural area. We get fresh and pure foods in the rural area. But in Dhaka it is not easy to get fresh and pure foods. The taste of food in the village is better than Dhaka City. In the village the quantity of cooked food is much bigger than here."* (Reshmi, 5 months pregnant)

What appeared to be the central issue surrounding the quantity of food that could be accessed and, consequently, the level of food insecurity experienced by the participants in Dhaka compared with the rural area, was the fact that rural people could be somewhat self-sufficient regarding food acquisition. Whereas in Dhaka, the women believed this was not something that could be done and therefore everything must be purchased:Furthermore, due to insufficient incomes, all the women accessing the local delivery centre’s antenatal services could not fully follow the advice given them from this biomedically-based service, particularly when it came to nutritional advice. Most respondents expressed the sentiment that '*poor men cannot afford’* things like '*milk and fish everyday’* (Jasmine, 7 months pregnant).

"*At that time there was a huge kitchen garden. We could get vegetables as much as needed. We had ponds. At that time nothing was bought. But it is not possible in Dhaka City. Here you have to buy everything and nothing is free of cost*." (Parul, 4 months pregnant)

"*[NGO] doctor advised me to take sufficient vegetables, milk and eggs…I cannot afford to manage those. I cannot even afford these once a month because of my financial crisis.*" (Mokta, 3 months pregnant)

#### Women’s shrinking space to save money

Women who undertook household food purchasing, and thereby had access to money daily, reported that they saved leftover *bazaar* money after making the daily food purchases. This money was deemed by all the women as "*security money"* and was used to mediate times of scarcity. Women reportedly used these savings to subsidize the cost of food if husbands were unable to earn enough in the day.

*'I also save some portion of money from the bazaar. Sometimes my husband cannot earn. At that time I spend this saved money. My husband also knows about this…[the amount I save] depends on the price of the bazaar items. When the price is high I save 10 taka* (about 0.14 CAD)*. When the price is low I can save 15 to 20*.’ (Rokeya, 8 months pregnant)

While this practice of saving leftover bazaar money was widespread and beneficial for women in accessing food, due to the rising costs of food women reported they were less and less able to save the small amounts of leftover daily *bazaar* money. To be able to continue saving leftover *bazaar* money despite rising costs of food, women reported that they purchased less food:

*"I sometimes buy a less amount of oil and rice. By this process I can still save a little amount of money…Nobody taught me this strategy. I learnt it from experience in need."* (Mony, 8 months pregnant)

One possible reason women attempted to continue to save money in spite of rising food prices and inadequate incomes was because it appeared, from the women’s demeanours when discussing this topic, to be an 'empowering’ practice that was not talked about openly with their husbands although their husbands were aware it was occurring. With amusement, women discussed this topic as an *'open secret’* and a practice that was undertaken by *all* women responsible for the daily food purchasing. Consequently, when women start purchasing less and less food due to the rising costs of food to save a little bit extra from the daily *bazaar* money, their nutritional status could be affected.

## Discussion

The aim of this study was to explore the lived experiences of food insecurity and household food provisioning strategies amongst ultra-poor, pregnant women in urban Bangladesh. The results showed that, although women had the knowledge of healthy pregnancy eating practices and more relaxed gender rules which allowed them physical and social access to foods sold at the *bazaar*, they were unable to access sufficient amounts and types of food due to rising food prices.

The widespread knowledge of what constitutes healthy pregnancy eating practices is supported by the literature showing women in the Dhaka *bastees* have an accurate idea of the biomedically-defined root causes of malnutrition [60] and also that they are aware of the inadequacy of their families’ diet [26]. Valliantos [56] concluded that due to the growing presence of maternal health programs, participants’ definition of a healthy diet while pregnant was exactly the same as what she terms the "mantra" of the NGO fieldworkers in her fieldsite: "eat green leafy vegetables, milk, juice and fruits". Interestingly enough, this "mantra" was also recited by the participants in this study. Women living in urban slums and who are engaged in work outside the home are likely being exposed to new sources of knowledge, like garment co-workers or bosses, and having fewer elders around to ensure compliance to traditional beliefs. While nutritional education has been important in increasing knowledge of healthy eating during pregnancy, there is likely no need to provide increased efforts towards nutritional education for pregnant women [26,56,60].

Every domain has multiple knowledge systems contained within it [61] and the domain of pregnancy and childbirth is no exception. In the literature, this domain is associated with two knowledge systems: the biomedical and the traditional. Both of these knowledge systems have ways and rhetoric to explain the childbirth experience [62] including food practices. Inherent in privileging biomedically–orientated knowledge is the inevitable dismissal of other kinds of knowledge [63]. While participants largely did not report engaging in traditional food practices, there is the possibility that there was a discomfort in talking to the research team about more traditional practices for fear of judgement. This was found to be the case in ones study from rural Bangladesh [12].

Women in this urban field-site were experiencing a relaxation of traditional hegemonic gender roles. Women seemed to have increased control over household resources and increased mobility and visibility. For instance, many women were actively involved in the household management of finances and being responsible for household food purchases. The young married women in this study were mostly living either in nuclear families or were close to their natal kin, a phenomenon which has been reported in other urban immigration studies [57-59].

From observations, they appeared to be in a slightly less vulnerable position regarding "nutritional adequacy"; corroborating research that women’s health status is associated with the amount of contact she has with her natal kin [64] and that natal relatives in slums are the social connections that largely help women with economic problems, including accessing food [59,65]. That we observed gender norms relaxing in this study is supported by Salway *et al*. [15], who argued that the gender roles of women in Dhaka’s slums are in a "state of flux" (p. 346). This study supports their conclusion that increased visibility, mobility and control over resources may allow for an increased amount of opportunities and negotiation space for improving the terms of women’s lives. The authors do, however, suggest that these changes may not be welcome by women. Our findings support this suggestion as women in our study unanimously disliked doing the daily shopping and some continued to believe that these types of activities (*ie.* being out in public in general, being out in public while pregnant) were shameful.

Intra-household food allocation, the subject of multiple studies from Bangladesh and other low-income countries [16-18] was only reported by one participant to be an issue in this field site. As stated previously, the non-reporting of this issue may be due to the young age of our respondents, living in nuclear families, being pregnant with their first child, or the presence of a research team and the accompanying fear of being judged. Further research is needed to examine intra-household food allocation in this evolving and dynamic setting.

However, this study suggests that the new "power" women have acquired - knowledge of healthy eating and a relaxed gender order - is negated by increasingly high costs of food in urban Bangladesh. Despite the advantages provided by their urban location, they will perhaps remain as nutritionally deficient as their rural sisters, except the underlying causes are different. Nutritional deficiencies in rural women are more likely to be grounded in power structures of gender, whereas the urban women’s nutritional deficiencies may be grounded more in global power structures that are responsible for rising food prices and inadequate incomes. Women having more control over resources in the home (*e.g.,* having control over the resources to purchase food and the decision-making power to make food purchases) is necessary for women’s empowerment. Nevertheless, our research suggests this type of control is not sufficient and is limited by existing economic structures (*e.g.,* reliance on market-based food systems in urban areas).

Rural to urban migrations in Bangladesh due to the shifting global economy to more liberalized and globalized policies are proving to be largely detrimental for unskilled migrants [58]. In the current study, although family members and women were able to find employment, incomes were inadequate for confidently assuring that daily basic needs like food and shelter were met. Amartya Sen [66] demonstrated in his Nobel Prize awarding winning work that food security is first and foremost an issue of accessibility rather than availability. In the urban areas, food is available but, for poor, pregnant women who must wrestle with the global food price crisis amidst vastly insufficient incomes, not easily accessible. Some believe the current global food crisis is due to a decrease in food supplies as a result of decreasing arable land, climate change, peak oil, *etc.* (*i.*e., classic demand and supply economics) [67]. In the slums of Bangladesh, food in adequate amounts is still available for purchase (if one has the means). Access to this food is related to one’s ability to physically, socially and financially acquire food. The financial inability to acquire food due to increasing food prices, compounded by chronically inadequate incomes, was the most critical barrier for the participants in this study. As well, the market dependence for food in urban areas further increases their vulnerability to globally rising food prices [68].

### Study limitations

One limitation of this qualitative study is that the research was limited to one site in urban Bangladesh, raising questions around generalisabiltiy. However it has provided an in-depth description of the lived experiences of ultra-poor pregnant women in urban Bangladesh. There is also the question of whether knowledge of healthy eating and a relaxed gender order actually lead to improved nutritional status. This study did not quantitatively measure these associations and more research surrounding these questions is needed. Another important limitation in the data was that, despite the RA being the same gender and nationality as the pregnant participants, the socioeconomic difference between them still contributed to an inevitable, already-existing power differential created by the presence of the PI, a westerner. This power differential may have influenced the information given the research team by the participants. Thirdly, as all interviews were undertaken and recorded in *Bangla*, the PI relied on translated transcripts and the cultural interpretations of the RA leading to potential biases in the interpretation. Lastly, as is standard with qualitative research, these findings cannot necessarily be applied outside of this particular context.

## Conclusion

In the formulation of new development goals addressing maternal and child health as well as malnutrition, it will become increasingly more crucial to consider that poor, urban women are experiencing rising levels of food insecurity due to rising costs of food, insufficient incomes and their perceived reliance on the market-system. This larger and more threatening influence on women’s access to food may be negating the improvements in knowledge and women’s mobility, visibility, and decision-making in her household which were observed in this study as well as a number of other South Asian studies. Unfortunately, while improving incomes amongst the ultra-poor in Dhaka may be a feasible approach to improving nutritional statuses, it has proven to be an arduous battle. This is evidenced by occurrences such as repeated violent strikes by garment workers justifiably decrying unacceptably low wages which has led to a wage increase of almost 80% since 2010, up to 40 dollars a month[69]. Also, while government-mediated food-entitlements have been suggested by scholar-activists like Amartya Sen [66], they have been criticised by others like Vandana Shiva[70], a world renowned Indian physicist cum eco-feminist activist. While entitlements to food are necessary to improve nutritional statuses and food security, they are not sufficient and they must occur free from corruption and as a supplement to other activities that address the root causes of hunger [70,71]. Rising food prices are rooted in systemic, global structures and ideologies which are not usually subject to external pressure to change. Improvements will have to emerge from the ground up; improving the basics like overall education, sanitation and water quality, medical care, and devising novel ways to improve household food provisioning strategies in the context of urban poverty. The creators of the up-and-coming Sustainable Development Goals [44] must also consider engaging with the larger global systems, like the food system, that have to-date stifled the achievements of Millennium Development Goals around the world.

## Abbreviations

BMI: Body Mass Index; NGO: Non-Governmental Organization; PI: Primary Investigator; RA: Research Assistant; HREB: Human Research Ethics Board.

## Competing interests

The authors declare that they have no competing interests.

## Authors’ contributions

AL conceived and undertook the study under the supervision of ZM, SR and NW. AL and ZM drafted the manuscript. All authors were involved in critically revising the manuscript for important intellectual content. All authors read and approved the final manuscript.
